# Talin is a substrate for SUMOylation in migrating cancer cells

**DOI:** 10.1016/j.yexcr.2018.07.005

**Published:** 2018-09-15

**Authors:** Zhiyao Huang, Diana Barker, Jonathan M. Gibbins, Philip R. Dash

**Affiliations:** School of Biological Sciences, University of Reading, Reading, Berkshire RG6 6UR, United Kingdom

**Keywords:** Talin, SUMOylation, Focal adhesions, Cancer cell migration

## Abstract

Focal adhesions (FAs) play an important role in cancer cell migration and metastasis by linking the actin cytoskeleton to the extracellular matrix, allowing the cell to generate traction. SUMOylation is a post-translational modification of proteins on lysine residues that can affect protein localisation, turnover and protein-protein interactions. In this study, we demonstrate that talin, a key component of FAs, can be post-translationally modified by SUMOylation in MDA-MB-231 breast cancer cells and U2OS osteosarcoma cells. Furthermore we demonstrate that SUMOylation regulates the dynamic activities of FAs including their number, size and turnover rate. Inhibiting SUMOylation significantly reduced the speed of cell migration. The identification of talin as a SUMO target provides insight into the mechanisms regulating focal adhesion formation and turnover and potentially identifies a novel mechanism underlying cell migration.

## Highlights

• Talin is SUMOylated in cancer cells.
• SUMOylation may regulate focal adhesion disassembly and cell migration.
• SUMOylation may also regulate cleavage of talin.
• Other focal adhesion proteins have been identified as potential SUMO targets including filamin-A and vinculin.

## 1 Introduction

SUMO (small ubiquitin-related modifier) family proteins are ubiquitin-related small proteins, which are ~15 kDa and can be conjugated to cellular substrates on lysine residues in an analogous way to ubiquitin [1]. This type of post-translational modification, known as SUMOylation, is implicated in the control of a wide variety of cellular processes, such as cell signalling, cell cycle and nuclear modification [2]. The SUMO family consists of at least three isoforms – SUMO 1 is mainly found in the nucleus while SUMO 2 and 3 share 95% homology and are generally considered together as SUMO 2/3, and are mainly located in the cytoplasm [3]. SUMO 1 and SUMO 2/3 proteins work closely with SUMO proteases and conjugating enzymes in the SUMOylation cycle [4]. Protein substrate modification with SUMO relies on a single E2 ubiquitin conjugating enzyme, Ubc9, in the SUMOylation pathway, where Ubc9 is unique among E2 enzymes in its capability to specifically recognize and conjugate SUMO1 or SUMO 2/3 to their substrates [5], [6].

Focal adhesions (FAs) are large multi-protein complexes that play a central role in cell migration by linking the extracellular matrix (ECM) bound to transmembrane integrin molecules with the actin cytoskeleton, allowing the cell to generate traction [7]. Cell migration on and through ECM requires the turnover of focal adhesions [8] so that FAs form when the cell attaches to ECM, generate traction allowing the cell to move forward and then disassemble to allow new FAs to form at the leading edge of the cell [9], [10], [11]. Rapid and dynamic FA assembly and disassembly processes are controlled and regulated spatiotemporally at the leading edge and the rear end of the migrating cell and are required for successful cell migration [12], [13]. More than 150 FA or FA-associated proteins have been identified [14] with some of the most important FA proteins for cell migration including focal adhesion kinase (FAK), talin, vinculin, paxillin and zyxin [15], [16]. Previous studies have shown that FAK interacts with PIAS1, which promotes the FERM domain of FAK to be covalently modified by SUMO-1 at the ε-amino position of lysine 152 enhancing its autophosphorylation [17]. SUMOylation of FAK occurs mostly in the nucleus and possibly independently of cell adhesion as PIAS1 is predominantly a nuclear protein, suggesting that cytoplasmic FAK may undergo nucleocytoplasmic cycling. Inhibiting protein SUMOylation after 6 h of ginkgolic acid (GA) treatment (an inhibitor of SUMOylation) in HEK293T cells resulted in a significant decrease in SUMO1 and SUMO 2/3 conjugation and a reduction in Tyr-397 phosphorylation in the SUMOylated form of FAK; FAK was therefore identified as a substrate for SUMOylation [17], [18], [19] but no other focal adhesion proteins have subsequently been shown to be modified by SUMOylation. In addition, no studies have shown SUMO modification of proteins within focal adhesions.

In this study, we show that talin, a key component of focal adhesions is a SUMO substrate. Talin is required in FAs for linking integrin to actin filaments and, together with kindlin, it is important for inside-out integrin activation, which can relay the inside-out signals to maintain an activated integrin state at the ECM-substrate surfaces [20], [21], [22], [23]. We demonstrate that talin localised in FA's is SUMOylated and also show that inhibiting SUMOylation significantly increased the number and size of talin-containing FAs as well as reducing their turnover rate and decreasing the speed of cell migration. We also propose that SUMOyltion of talin may regulate talin cleavage by calpain, an important regulator of FA disassembly and turnover. We have therefore have identified a potential new role for SUMOylation in the regulation and function of talin which could be important in cell migration.

## 2 Results

### 2.1 Blocking SUMOylation increases the number, size and turnover time of focal adhesions and reduces the speed of cell migration

**Fig. 1:**
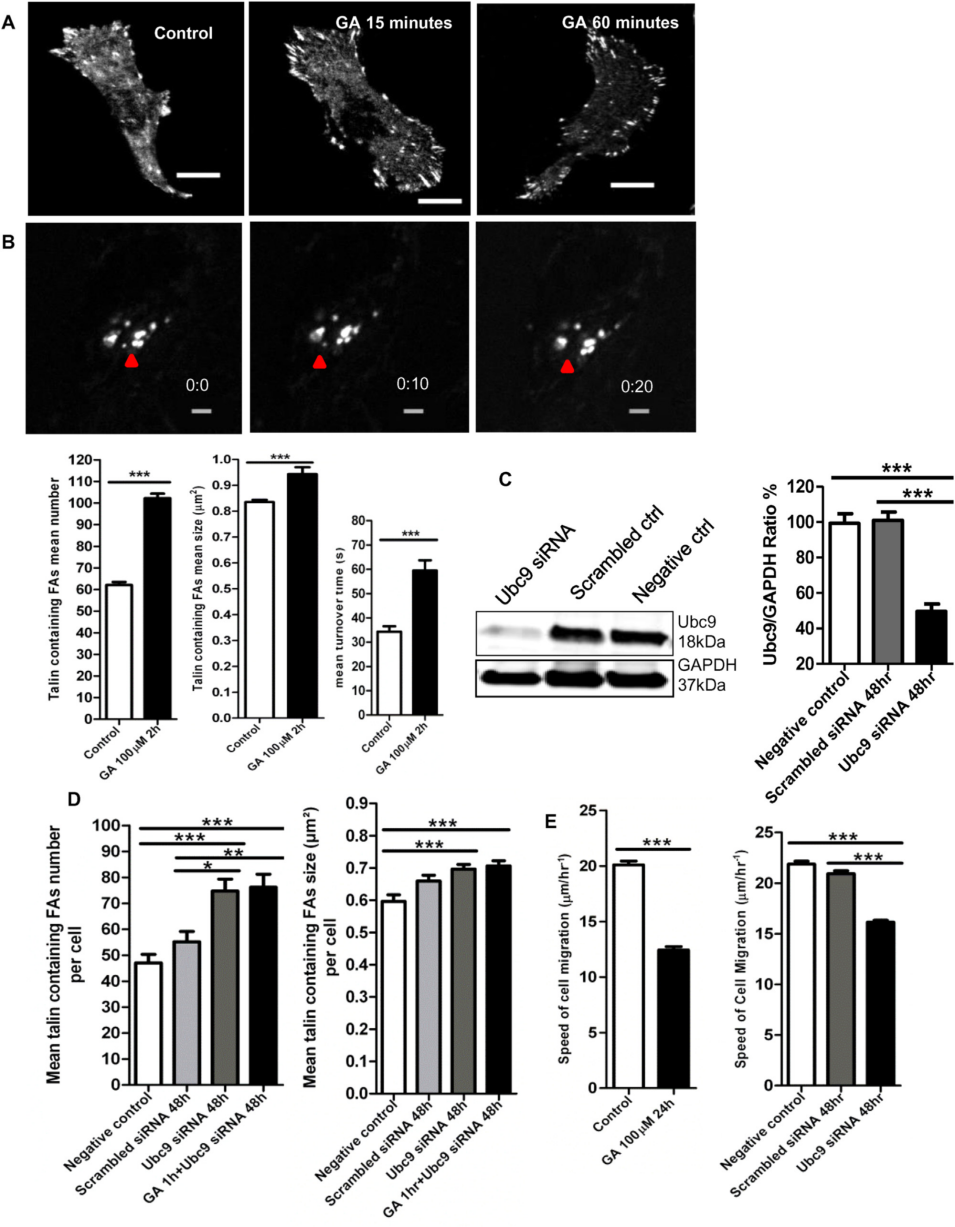
Inhibiting SUMOylation increases the number, size and turnover of focal adhesions and reduces the speed of cell migration in MDA-MB-231 cells. 1A. MDA-MB-231 cells were grown on 0.2% gelatin-coated glass coverslips. Immunostaining of talin containing FAs were shown in the control or after 15 or 60 min of 100 µM GA treatment (scale bar = 20 µm). 1B. MDA-MB-231 cells were grown on top or at the edge of 2 mg/ml rat tail collagen I (scale bar = 10 µm). GFP-talin containing FAs were shown in untreated live MDA-MB-231 cells establishing the dynamic turnover of FAs. The red arrow indicates a single talin-containing FA was turning over in 10 s, initially appearing at 0 s, continuing to be present for 10 s and disappearing at 20 s. In this live-cell GFP-FA turnover assay, 2 h of 100 µM GA treatment increased the mean number, size or turnover time of talin containing FAs (n = 5, individual replicates, data shown as mean ± SEM, p < 0.0001 ***, two-tailed unpaired *t*-test, >300 cells were counted either for the control or the GA treatment in the mean number and size of FAs; for turnover rate, 280 or 182 adhesion number was counted for the control or the GA treatment manually). 1C. 48 h of 25 nM Ubc9 siRNA treatment caused knockdown of Ubc9 E2 enzyme, the bar chart is presented as Ubc9 vs. GAPDH ratio (n = 4, individual replicates, data as mean ± SEM, p < 0.0001 ***). 1D. MDA-MB-231 cells were grown on 0.2% gelatin-coated glass coverslips. 48 h of 25 nM Ubc9 siRNA treatment increased the mean number or size of talin containing FAs (n = 4, individual replicates, data as mean ± SEM, p < 0.0001 ***, one-way ANOVA with post-hoc test, 255, 173 and 240 cells were counted for negative control, scrambled siRNA or Ubc9 siRNA treatment); although there was no further increase in the combination treatments using GA with Ubc9 siRNA together. 1E. MDA-MB-231 cells were grown in 2 mg/ml rat-tail collagen I. 24 h of 100 µM GA treatment decreased the speed of cell migration significantly (n = 3, individual replicates, mean ± SEM, p < 0.0001 ***, two-tailed unpaired *t*-test, 361 or 273 cells were counted for the control or the GA treatment). 48 h of 25 nM Ubc9 siRNA decreased the speed of cell migration significantly (n = 4, individual replicates, mean ± SEM, p < 0.0001 ***, one-way ANOVA with post-hoc test, 550 > cells were analysed either for the control or the siRNA/scrambled siRNA treatment).

The properties of FAs were investigated whilst inhibiting SUMOylation with ginkgolic acid (GA), an E1 inhibitor that can prevent SUMOylation of target proteins [24]. MDA-MB-231 breast cancer cells were grown on glass coverslips (Fig. 1A) or 2.5D collagen (at the collagen/plastic interface or on top of collagen) (Fig. 1B). The mean number, size and turnover time of FAs were analysed for the cells on collagen after GA treatment (Fig. 1B). Two hours of 100 µM GA treatment significantly increased the mean number of talin containing FAs per cell from 62 ± 1 to 102 ± 2, the mean size of FAs increased from 0.835 ± 0.009 µm^2^ to 0.944 ± 0.027 µm^2^ and the turnover time increased from 34.3 ± 2.29 s to 59.5 ± 4.24 s, respectively (p < 0.0001***, Fig. 1B). Ubc9 siRNA was used to target the E2 Ubc9 enzyme. MDA-MB-231 cells were transfected with 25 nM Ubc9 siRNA or 25 nM scrambled siRNA for 48 h (Fig. 1C). Following siRNA treatment the Ubc9 E2 enzyme expression significantly decreased from 101.0 ± 4.65 (Ubc9/GAPDH ratio x 100) to 49.6 ± 4.19 (p < 0.0001***); whereas the scrambled siRNA control did not cause significant knockdown of Ubc9 expression (ns, p = 0.82). Treatment of cells with Ubc9 siRNA for 48 h significantly increased the mean number of talin-containing FAs per cell from 47 ± 3–75 ± 5 and their mean size from 0.596 ± 0.021 µm^2^ to 0.697 ± 0.014 µm^2^ (p < 0.0001***, Fig. 1D). Combinations of GA and Ubc9 siRNA did not produce an additional increase in the mean number (ns, p = 0.83) or size of the FAs (ns, p = 0.66) compared to Ubc9 siRNA treatment alone (Fig. 1D). Treatment of cells with 100 µM GA for 24 h significantly reduced the mean speed of cell migration from 20.1 ± 0.341 µm/h^−1^ to 12.4 ± 0.306 µm/h^−1^ (p < 0.0001***, Fig. 1E). Treatment with Ubc9 siRNA for 48 h also significantly decreased the mean speed of cell migration from 21.9 ± 0.299–16.2 ± 0.186 µm / h^−1^ or from 20.9 ± 0.295 to 16.2 ±0.186 µm / h^−1^ (p < 0.0001***, Fig. 1E).

### 2.2 Talin is SUMOylated in MDA-MB-231 cells

**Fig. 2:**
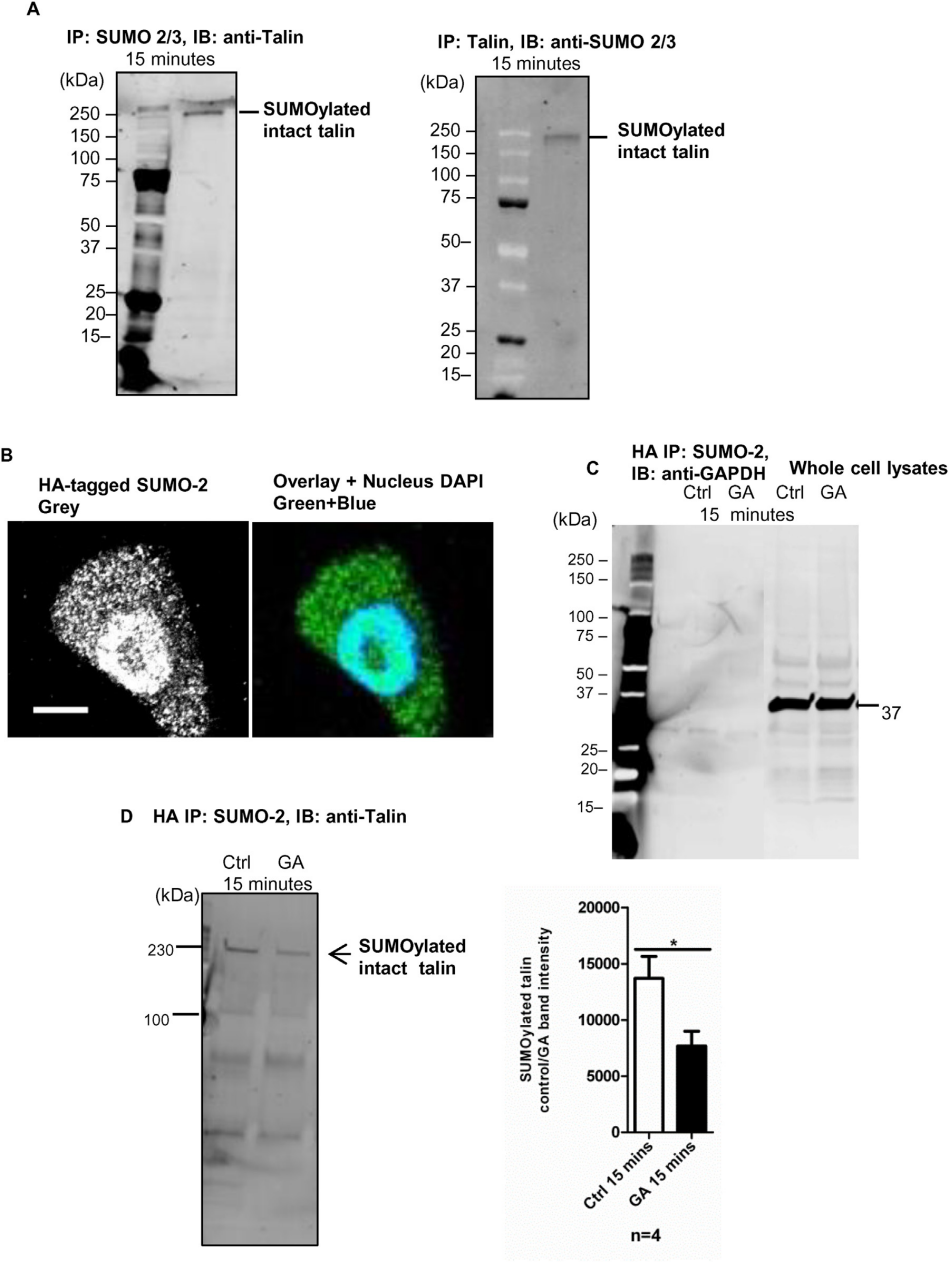
Talin is a substrate for SUMOylation. 2A. SUMO 2/3 was immunoprecipitated and the subsequent samples separated by SBS-PAGE and probed with a talin antibody after Western blotting. This showed that talin could be SUMOylated at 250 kDa; similar conclusions were reached following the reverse IP (n > 3, repeated more than three individual experiments). 2B. SUMO-2 expression in MDA-MB-231 cells (greyscale); DAPI was for nucleus staining. Overlayed image indicates SUMO-2 expression in the nucleus and in the cytoplasm (scale bar = 20 µm). **2C.** HA-SUMO-2 IP pulldown showed that GAPDH was not SUMOylated in MDA-MB-231 cells. This acted as a negative control for the HA-SUMO-2 pulldown IP. 2D. HA-SUMO-2 IP pulldown showed that talin could be SUMOylated (230 kDa, a different antibody was used) (n = 4, individual replicated experiment). After 15 min of 100 µM GA treatment, the band intensity for the SUMOylated talin was significantly reduced (n = 4, mean ± SEM, p = 0.043, two-tailed unpaired *t*-test).

SUMO 2/3 was immunoprecipitated from whole cell lysates and an N-terminus talin-1 antibody was used to probe for the presence of talin in the IP SUMO 2/3 samples. Western blotting revealed the presence of talin in the SUMO2/3 immunoprecipitates suggesting that talin is a substrate for SUMOylation (Fig. 2A). The reverse IP (immunoprecipitating talin and probing the resulting western blot with a SUMO2/3 antibody) also confirmed this finding (Fig. 2A). HA-SUMO-2 transfection showed SUMO-2 was mostly present in the nucleus and the cytoplasm of MDA-MB-231 cells (Fig. 2B). GAPDH was detected in the cells but there was no evidence that GAPDH was present in the HA-SUMO-2 IP pulldown, therefore GAPDH is likely not a substrate for SUMOylation (Fig. 2C). The HA-SUMO-2 IP indicated the full-length talin at 230 kDa (detected using a different talin c-9 antibody) was SUMOylated (Fig. 2D). The band intensity of the SUMOylated talin was significantly reduced from 13,700 ± 1954 to 7684 ± 1313 after 15 min of 100 µM GA treatment (p = 0.043, Fig. 2D).

### 2.3 Full-length and cleaved talin is SUMOylated in isolated focal adhesions

**Fig. 3:**
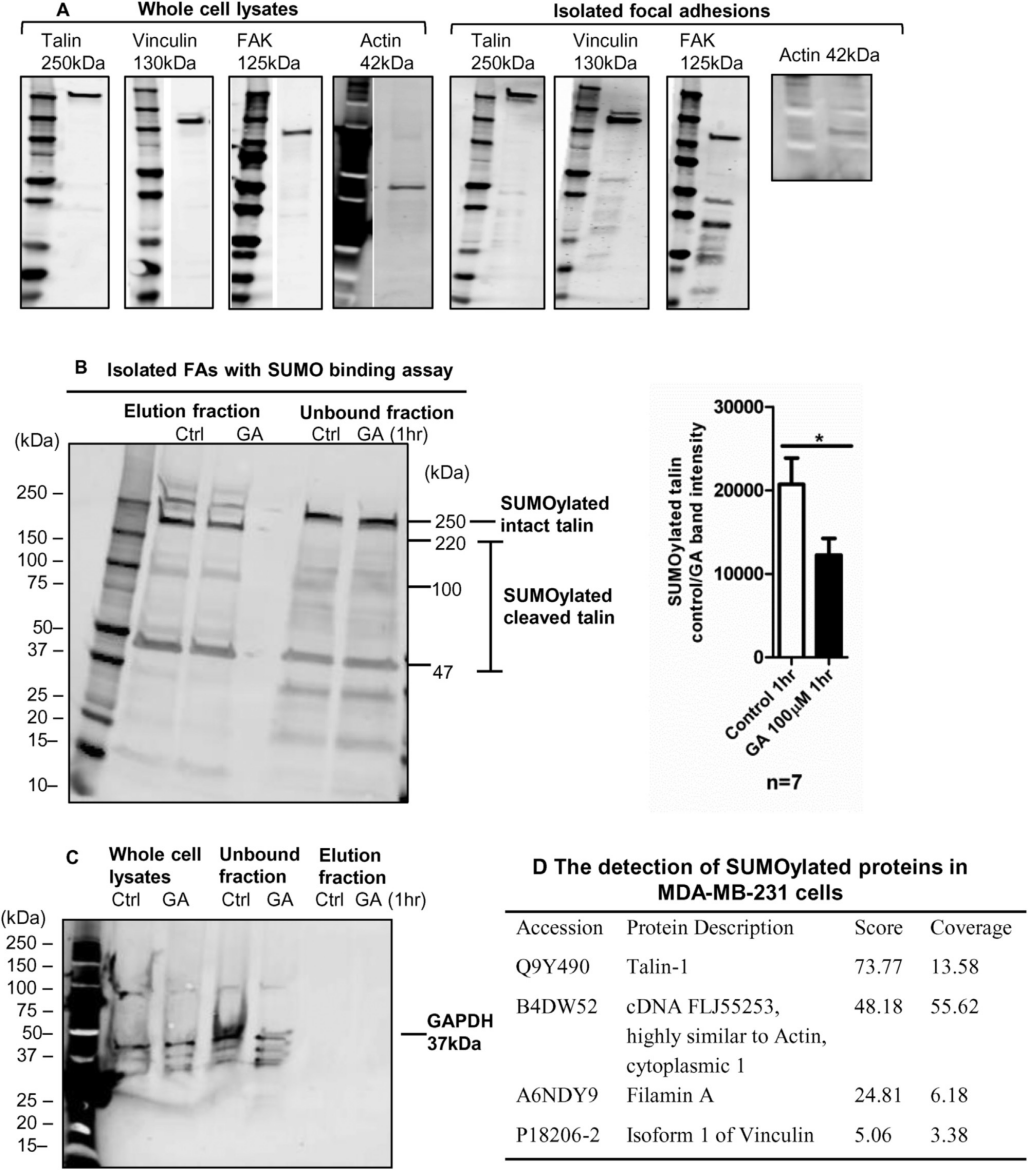
Talin is SUMOylated in isolated focal adhesions and is cleaved in concentrated isolated FAs. 3A. Western blotting showed that the main FA proteins were present in the isolated FAs as well as in the whole cell lysates as talin, vinculin, FAK and actin. 3B. The isolated FAs were used in the SUMO binding assay which showed that talin was a substrate for SUMOylation at 250 kDa. Additionally, talin seemed to be cleaved. The 220 kDa fragments of talin were found in the eluted fraction but not in the unbound whole cell lysate fraction. The fragmented talin could be SUMOylated (n = 3, individual replicated experiment). After 1 h of 100 µM GA treatment, the band intensity for the SUMOylated talin was significantly reduced (n = 7, mean ± SEM, p = 0.036, two-tailed unpaired *t*-test, individual replicated experiment, data was combined from the HA-SUMO-2 IP pulldown and the SUMO binding assay with FAs). 3C. GAPDH was not detected as SUMOylated using the SUMO binding assay (n = 3, individual replicated experiment). 3D. The cells were incubated with the SUMO matrix-containing columns in the SUMO binding assay to obtain eluted SUMOylated proteins. These were reserved in 0.1% formic acid previously and sent for mass spectrometry LC-MS/MS analysis. Talin, filamin A, actin and vinculin were detected as SUMOylation substrates.

To investigate if talin could be SUMOylated within FAs, the FAs were first isolated from the cells. The method of isolating FAs was adapted from Kuo et al. [25], [26]. Isolated FA samples were used in the VIVAbind™ SUMO assay. In the unbound fraction (non-SUMOylated) from this assay talin, vinculin, FAK and actin were detected in both whole cell lysates and isolated FAs, suggesting that the FA isolation method was successful (Fig. 3A). Full-length talin (250 kDa) was detected in the eluted (SUMOylated) fraction, suggesting that talin was SUMOylated in the isolated FAs (Fig. 3B). Similarly, fragments of talin (detected at 220 kDa, 100 kDa and 47 kDa) were also found. The 220 kDa fragment of talin was not detected in the unbound fraction (Fig. 3B) suggesting that it is only the SUMOylated form of talin that is cleaved. The band intensity of SUMOylated talin from the eluted fraction was reduced significantly from 20,710 ± 3157 to 12,250 ± 1984 after 1 h of 100 µM GA treatment (p = 0.036, Fig. 3B). The samples were also examined for the presence of GAPDH as a negative control. GAPDH was present in the whole cell lysates and unbound (non-SUMOylated) fraction, but it was absent after the elution during the SUMO binding process (Fig. 3C). Talin was also detected as a substrate for SUMOylation in mass spectrometry analysis of the eluted fraction from isolated FAs along with other potentially novel SUMO substrates of filamin A, actin 1 and vinculin (Fig. 3D).

### 2.4 Inhibiting SUMOylation does not affect the total expression of talin or FAK

**Fig. 4:**
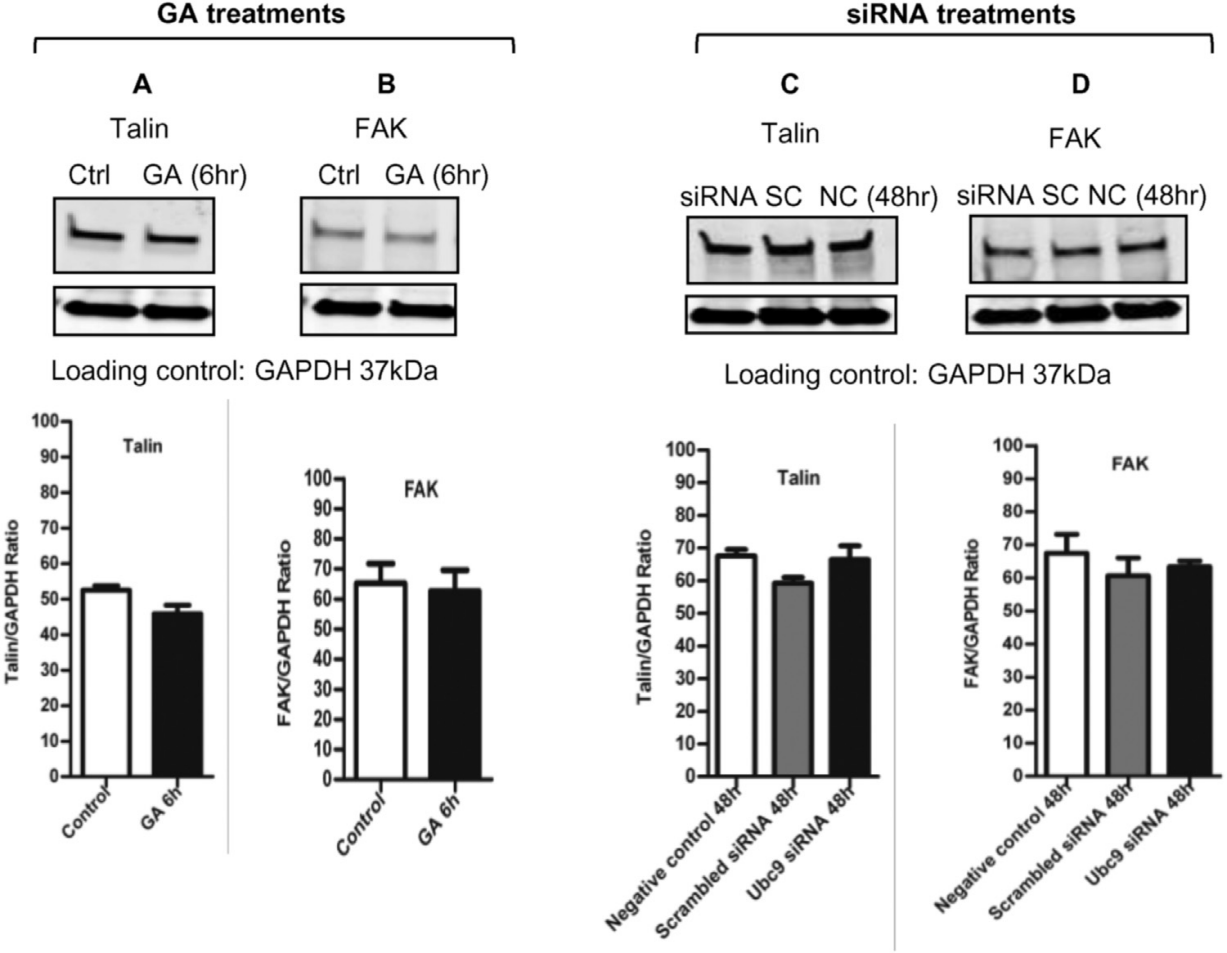
Inhibition of SUMOylation does not alter expression of talin for FAK. 4A-B. 6 h of 100 µM GA treatment in MDA-MB-231 cells did not change total cellular talin or FAK expression in the western blots (n = 3, individual replicated experiment). 4C-D. 48 h of 25 nM Ubc9 siRNA treatment in MDA-MB-231 cells did not change total cellular talin or FAK expression in the western blots (NC: negative control, SC: scrambled control, n = 3, individual replicated experiment).

Since one possible role of SUMOylation is to prevent ubiquitination of lysine residues and therefore prevent proteasome mediated protein degradation we investigated whether inhibition of SUMOylation would cause a decrease in cellular levels of talin or FAK. Treatment of cells with 100 µM GA for six hours did not affect the total expression of talin or FAK in MDA-MB-231 cells (Fig. 4A and B). Similarly, 48 h treatment with 25 nM Ubc9 siRNA did not affect talin or FAK protein levels in the cells (Fig. 4C and D). These results suggest that the role of SUMOylation of these FA proteins is independent of the effects of SUMOylation on protein stability.

### 2.5 Inhibiting SUMOylation affects formation and function of FAs in U2OS cells

**Fig. 5:**
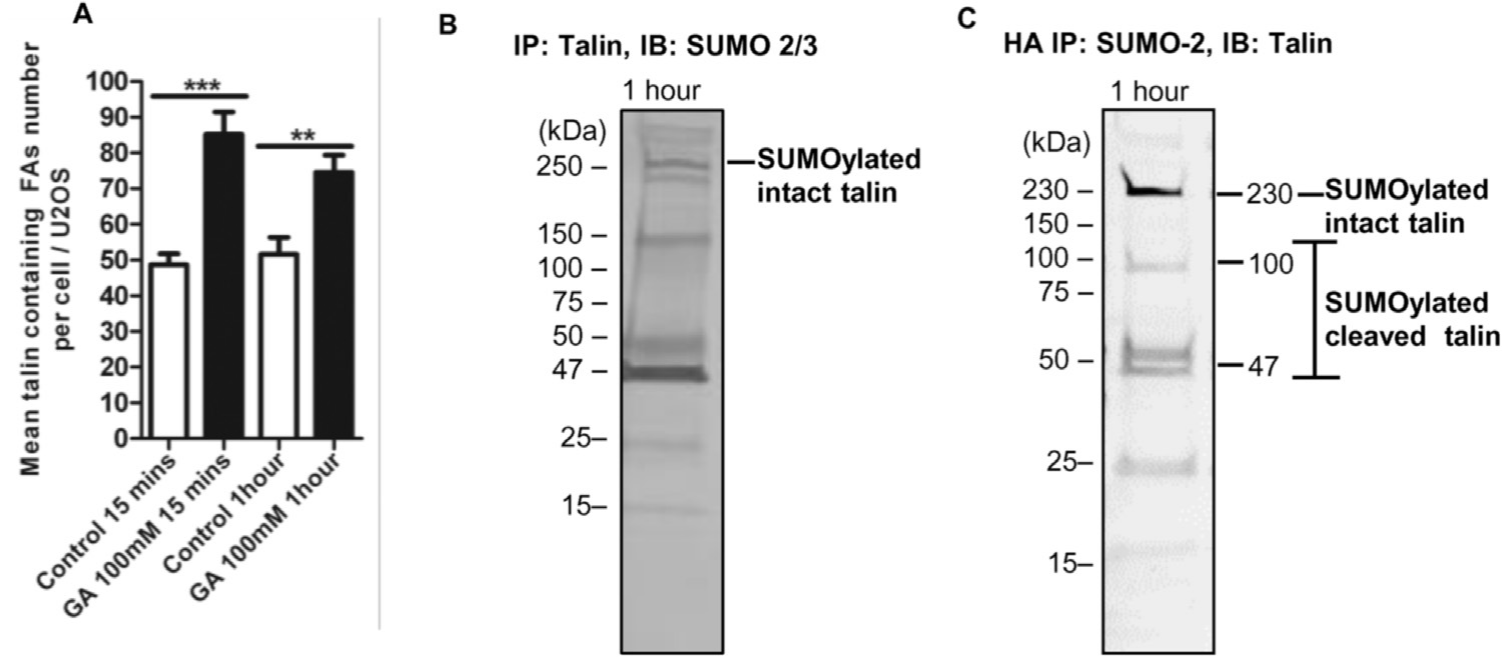
Inhibition of protein SUMOylation in U2OS cells. 5A. U2OS were grown on 0.2% gelatin-coated coverslips. 15 min or 1 h of 100 µM GA treatment increased the mean number of talin containing FAs significantly (n = 3, mean ± SEM, GA 15 min: p < 0.0001***, GA 1 h: p = 0.0026**, one-way ANOVA with post-hoc test, 92 or 71 cells were counted for the control or GA 15 min treatment; 68 or 91 cells were counted for the control or GA 1 h treatment). 5B. Talin was a substrate for SUMOylation in U2OS cells (n = 4, individual replicated experiment). 5C. HA-SUMO-2 IP pulldown indicated that talin was SUMOylated (n = 3, individual replicated experiment).

An osteosarcoma cell line, U2OS, was used for comparison with the MDA-MB-231 cell line to determine whether the SUMOylation effects on FAs are found in other cells. Treatment of U2OS cells with GA for 15 min significantly increased the mean number of talin containing FAs per cell from 49 ± 3 to 85 ± 6 (p < 0.0001***, Fig. 5A). Treatment of U2OS cells with GA for 1 h significantly increased the mean number of talin containing FAs per cell from 52 ± 5 to 75 ± 5 (p = 0.0026**, Fig. 5A). Talin was also found to be SUMOylated in U2OS cells (Fig. 5B). To confirm talin SUMOylation U2OS cells were transfected with an HA-tagged SUMO-2 plasmid and talin was found to be SUMOylated in the HA-SUMO-2 IP pulldown (Fig. 5C). As with MDA-MB-231 cells, fragments of talin were also obtained (Fig. 5C) from the U2OS cells.

## 3 Discussion

Inhibiting protein SUMOylation using GA or Ubc9 siRNA increased the number and size of talin containing FAs in cells, suggesting that SUMOylation may play a critical role in the regulation of FAs. We also observed an increased turnover time of FAs following inhibition of SUMOylation suggesting that SUMOylation of FA proteins may lead to increased stability of FAs, impaired FA disassembly and therefore and increase in FA number and size. The combined effects of larger, more numerous and more stable FAs are that cell migration was significantly reduced. We therefore propose that SUMOylation may play an important regulatory role in FA disassembly.

Kadaré et al. identified FAK, a key FA protein, as a substrate for SUMO 1. However they also showed that SUMOylation of FAK occurred mostly in the nucleus and therefore may be independent of its role in cell adhesion and migration [17]. Given the presence of SUMOylated talin within FAs and the effects of SUMOylation inhibitors on FA turnover and size as well as on cell migration we believe that this study presents the first evidence of a direct role for SUMOylation in the regulation of focal adhesions. Previous studies indicate that only a small fraction of the SUMO substrates, often less than 1%, are SUMOylated at any given time [27]. It is possible that SUMOylation of talin occurs directly in the FAs with the majority of talin outside FAs existing in a non-SUMOylated state or alternatively talin may be SUMOylated in the cytosol and then cycled to the FAs in SUMOylated form when required. Since inhibition of SUMOylation leads to continued growth of FAs our data supports the hypothesis that talin is recruited into FAs in a non-SUMOylated form with SUMO conjugation likely to occur after talin has been recruited into the FA, possibly promoting the subsequent disassembly of the FA complex.

Talin is a 250 kDa molecule consisting of the ~50 kDa (47 kDa) globular N-terminal FERM (band 4.1, ezrin, radixin and moesin) domain (F1, F2 and F3 subdomains), which contains binding sites in β-integrin cytoplasmic regions; the C-terminal tail/rod domain of talin (220 kDa) contains several binding sites for vinculin, a second lower-affinity integrin binding site and at least two binding sites for actin [28], [29], [30], [31], [32]. Full-length talin was found to be SUMOylated in both MDA-MB-231 and U2OS cells as well as within isolated FAs in MDA-MB-231 cells. The function of talin in FAs is thought to be regulated by its cleavage by calpain [33], [34], [35], [36] and we observed that a small proportion of talin was cleaved in the migrating MDA-MB-231 cells. The cleaved fragments of talin were potentially SUMOylated suggesting that talin may be SUMOylated on multiple sites (Table S1).

In Table S1 and S2, two separate bioinformatics tools, SUMOplot and GPS SUMO, predicted that talin could be SUMOylated on lysines 2445 and 841, confirming the possibility that talin may be SUMOylated on multiple sites and suggesting avenues for future investigations into the mechanism through which SUMOylation acts on talin. Previous predictions of SUMO sites using SUMOplot and GPS SUMO have generally been borne out by experimental verification. Lysine 386 of p53 was predicted to be a SUMO conjugation site and this was experimentally validated [37]; lysine 284 and 68 were predicted and experimentally validated to be SUMO modified sites for actin [38]; interestingly, no predicted consensus motif was predicted for Rac1, which was in agreement with the finding that Rac1 was SUMOylated in the non-consensus sites within the polybasic region of Rac1 as the main location for SUMO conjugation [39]. Finally, lysine 152 was predicted to be a SUMOylation site in FAK and this was experimentally validated [17].

One of the mechanisms through which SUMOylation may act is by competing with ubiquitin for key lysine sites thus preventing proteosomal degradation of the SUMO target. We found that the expression of talin was not affected after inhibiting protein SUMOylation globally suggesting that SUMO conjugation does not act to prevent degradation of talin. It is possible that SUMOylation may instead have a role in regulating protein-protein interactions between talin and other FA proteins. This explanation for the mechanism of action of SUMOylation in FAs is especially likely as other FA components such as vinculin and filamin-A were predicted and experimentally shown to be SUMOylated (Fig. S2). Filamin-A was predicted as a SUMOylation substrate [2]. In our mass spectrometry analysis (Fig. 3D), filamin A and vinculin were also detected and therefore may also be novel SUMOylation substrates. Given that FAK, talin, vinculin and filamin-A could potentially all be SUMOylated and that these protein interactions are highly involved in FA turnover and additionally, in Fig. S2, inhibiting SUMOylation increased the number, size and turnover time of both FAK and vinculin containing FAs it is possible that the role of SUMOylation could be diverse during FA protein interactions and in the dynamic regulation of FAs in migrating cells.

Talin is also the major protein involved in the activation of vinculin, since talin co-localizes with vinculin in the focal complexes and with the majority of FA proteins including β3-integrin, talin, vinculin, FAK, paxillin, α-actinin, VASP and zyxin [40]. The role of SUMOylation in the regulation of FAs may be broader than just the SUMOylation of talin reported here or the SUMOylation of FAK reported previously [17], [18]. Since inhibiting SUMOylation increased the number, size and turnover time of FAs in both MDA-MB-231 breast cancer cells and U2OS osteosarcoma cells it is also possible that the role of SUMOylation in the regulation of FAs and cell migration is a wide phenomenon in other migrating cancer cells.

In conclusion, we have identified that SUMOylation may have a novel role in the regulation of talin formation and function, the regulation of FA turnover and in cancer cell migration. These may lead to the explorations of SUMOylation in other FA proteins and hence its broader impact in FA protein-protein interactions, cell signalling and cell migration.

## 4 Experimental procedures

### 4.1 Cell lines and cell culture

MDA-MB-231 (Human breast cancer cells, ATCC) cells were maintained in DMEM containing 1 g/L D-glucose, L-glutamine and pyruvate and supplemented with 10% (v/v) fetal bovine serum (FBS, Gibco) and 1% Penicillin/Streptomycin (10,000 units/ml penicillin, 10,000 µg/ml streptomycin, Gibco). U2OS (Human bone osteosarcoma cells, ECACC) were maintained in McCoy's 5 A (Modified) Media containing L-glutamine (500 ml, Gibco) and supplemented with 10% (v/v) FBS and 1% Penicillin/Streptomycin. The cells were cultured at 37 °C/5% CO_2_ humidified environment.

### 4.2 DNA plasmids

pcDNA3 HA-Sumo2 WT was a gift from Guy Salvesen, U.S.A. (Addgene plasmid # 48967); pGFP (C3)-Vinculin was a gift from Klaus Hahn, U.S.A. (Addgene plasmid # 30312); GFP-Talin 1 was a gift from Anna Huttenlocher, U.S.A. (Addgene plasmid # 26724). HA SUMO-2, GFP-vinculin and GFP-Talin 1 plasmids were bought from Addgene.

### 4.3 SUMOylation inhibitor

Ginkgolic acid C15:1 was purchased from Sigma-Aldrich (UK).

### 4.4 Plasmid and siRNA transfection

silencer^®^ select validated siRNA targeting gene UBE21 (Ubc9) was provided by ambion^®^ by life technologies™). The Sense sequence (5ˈ- 3ˈ) was: CCACCAUUAUUUCACCCGAtt and the Antisense sequence was: UCGGGUGAAAUAAUGGUGGtt. For RNAi experiments MDA-MB-231 cells were treated with 25 nM scrambled siRNA or 25 nM Ubc9 siRNA (prepared in 0.2 × diluted Lipofectamine^®^ RNAiMAX reagent, 25 nM). Experiments were conducted 24 and 48 h post-transfection with siRNA. For plasmid transfections 1 × 10^5^ MDA-MB-231 cells were transfected with 1 µg DNA using FuGENE HD transfection reagent (Promega) according to the manufacturers’ instructions. The media was replaced with fresh media after 8 h transfection and the cells were analysed 24 h after transfection.

### 4.5 Collagen coating, FA turnover assay and cell migration assay

2 mg/ml collagen matrices were prepared by mixing 4.41 mg/ml chilled rat tail type I non-pepsinized collagen in acetic acid (BD Biosciences) with DMEM 1 × (100 µl/ml, 10 ×), 15–20 µl of the 1 M NaOH per diluted 1 ml collagen solution and chilled culture media to make up to 2 ml. All the ingredients were mixed thoroughly on ice and 200–400 µl of the collagen mixture was added to the centre of glass bottom dishes (ibidi) forming a ‘ring patch’. The collagen gel was allowed to polymerize and solidify in 5% CO_2_/95% humidified air environment at 37 °C initially for 20–30 min. Cells were either seeded around the edge of the ‘collagen patch’ or directly within the collagen gel. In the former case cells were left in the incubator to attach and settle for another 20–30 min before fresh DMEM media was added to cover the set collagen matrix. Following an overnight incubation individual MDA-MB-231 cells were aligned ‘front-to-rear’ moving along the edge of the collagen patch; other cells had already migrated through into the 3D collagen matrix. The cells were transfected with the GFP-talin plasmid overnight and treated with 100 µM GA for 2 h. Cells were also embedded directly in collagen. For each well in a 12-well plate, 600–800 µl of the mixed collagen containing the cells was pipetted and spread fully across the well area. The 12-well plate was left in the incubator for 30 min for the collagen matrix to set. 2 ml DMEM media was added into each well and the cells were grown overnight in the 3D collagen matrix.

### 4.6 Immunocytochemistry, confocal microscopy and live-cell timelapse microscopy

cells were immunostained using mouse anti-talin 1 monoclonal antibody (N-terminus), Clone TA205, 1 mg/ml, MAB1676, Merck Millipore (1:100) and Alexa-Fluor 488 conjugated goat anti-mouse antibody (1:100). Timelapse movies of FA turnover were taken on a Nikon A1-R confocal microscope with an environmental chamber to set temperature at 37 °C with CO_2_ supply. Imaging of FA turnover in live cells was performed using the NIS Elements AR (Nikon) software and the Nikon Intensilight CHGFI lamp with the 100 × oil immersion lens with pinhole 1.2 on GALVANO mode. The images were taken in the 1024* format in the xyz plane; fast z-stack three-dimensional images were scanned; line averaging 4 × to 16 × was taken. Fluorescent filters include DAPI (405), GFP (488), Cherry (630) and Cy5 (640−750). The laser fast mode was turned on and used as ½ (2 scanning/second). Pixel saturation indicator was allowed maximal 1% saturation. Images/Timelapse movies were recorded at 2 s, 8 s or 10 s intervals. 488 green and 450 DAPI blue fluorescence channels were used. Live-cell imaging timelapse experiments were performed on a motorised Nikon Eclipse TiE inverted microscope with an environmental chamber and the temperature was set up to 37 °C with a CO_2_ supply. NIS Elements software and PlanApo 10 × DIC L lens were used to capture images every 20 min.

### 4.7 Immunoprecipitation and western blotting

immunoprecipitation was either prepared using endogenous whole cell lysates or with lysates from cells transfected with a HA-tagged SUMO-2 plasmid. MDA-MB-231 cells were grown on 0.2% gelatin coated T25 flasks. 250–300 µl cold Pierce^®^ RIPA buffer (25 mM Tris HCl pH 7.6, 150 mM NaCl, 1% v/v NP-40, 1% v/v sodium deoxycholate, 0.1% v/v SDS, Thermo Fisher Scientific) supplemented with 1 × protease inhibitor cocktail (PIC, Calbiochem^®^) and 50 µM N-Ethylmaleimide was added to the cells and a disposable cell scraper was used in the IP or reverse IP whole cell lysate samples. Protein A/G PLUS-Agarose IP beads (0.5 ml agarose in 2 ml PBS buffer with 0.02% azide, Santa Cruz) were used in the washing and centrifugation steps. The cells were also transfected with a SUMO-2 plasmid in T25 flasks. The same lysis buffer was used directly onto the cells. The purified mouse IgG1 anti-HA.11 epitope tag antibody (Biolegend, dilution as 1:100-150) was added to each cell lysate sample and all the tubes were mixed/rotated continuously overnight at 4 °C. The Pierce™ protein A/G magnetic beads solution (10 mg/ml in H_2_O containing 0.05% NaN_3_, Thermo Scientific) and a magnetic stand (Millipore) were used during washing. The first supernatant was kept. The samples were heated using 2% v/v SDS gel-loading buffer at 95 °C for 5 min. The eluted proteins were run on 4–20% Mini-PROTEAN^®^ TGX™ precast polyacrylamide gels (10-well, 50 µl, Bio-Rad). Following western blot analysis, protein bands were visualised using either a Typhoon fluorescence imager FLA 9500 (GE Healthcare) or using chemiluminescence. The following antibodies were used for immunoprecipitation and western blotting experiments according to the supplier's instructions. Mouse anti-UBC9 monoclonal antibody (C-12), 200 µg/ml, sc-271057, Santa Cruz Biotechnology, INC. (WB: 1:250-500). Rabbit anti-GAPDH antibody, 1 mg/ml, G9545, Sigma (WB: 1:1000). Donkey anti-goat IgG-HRP: 200 µg/0.5 ml, sc-2020, Santa Cruz Biotechnology, INC. (1:5000). Anti-mouse IgG-HRP, Sigma (1:3000). Anti-rabbit IgG-HRP, Sigma (1:3000). Purified mouse anti-HA.11 epitope tag monoclonal antibody, 1 mg/ml, 16B12, Biolegend (IP: 1:100-150). Anti-GFP mouse monoclonal antibody [9F9.F9] ab1218, Abcam (IP: 1:150-200). Mouse anti-talin 1 monoclonal antibody reacting with an N-terminal epitope in human talin between amino acids 139–433, Clone TA205, 1 mg/ml, MAB1676, Merck Millipore (IP: 1:100, WB: 1:1000). Mouse anti-talin monoclonal antibody (C-9, N-terminus), 200 µg/ml, sc-365875, Santa Cruz Biotechnology, INC. (WB: 1:200). Rabbit anti-talin polyclonal antibody (H-300, N-terminus), 200 µg/ml, sc-15336, Santa Cruz Biotechnology, INC. (WB: 1:200). Goat anti-SUMO 2/3 polyclonal antibody (N-18, N-terminus), 200 µg/ml, sc-26969, Santa Cruz Biotechnology, INC. (WB: 1:250-500). Rabbit anti-SUMO 2/3 polyclonal antibody detecting at the N-terminus, 0.5 mg/ml, P61956, Millipore (WB: 1:1000). Rabbit anti-SUMO-2 (Sentrin-2) polyclonal antibody, 519100 Invitrogen (WB: 1:1000). Alexa-Fluor 647 conjugated donkey anti-goat antibody (Cy5, 1:1000). Alexa-Fluor 546 conjugated donkey anti-rabbit antibody (Cy3, 1:1000).

### 4.8 Isolation of focal adhesions

the FA isolation protocol was modified from Waterman C.M. (2011). MDA-MB-231 cells were grown on 15 µg/ml fibronectin-coated T25 flasks or petri dishes and incubated at 37 °C/5% CO_2_ for 1–2 days. The cells were incubated in 2.5 mM low ionic strength TEA buffer (Sigma, pH 7.0) for 3 min at room temperature. An Ultra Waterpik^®^ Waterflosser^®^ Jet water flush was used to give strong trituration pressure for 10 s to remove the nuclei, cell bodies, soluble proteins and materials of the cytoplasm with the water tank filled with 20 ml of 1 × flushing buffer (1 × PBS mixed with 1 × PIC and 50 mM NEM). The isolated FAs were collected in denaturing lysis buffer and sonication was supplied to the collected FA samples for 10 s on ice.

### 4.9 VIVAbind™ SUMO assay

the cells were collected after isolation of their focal adhesions in the Pierce^®^ RIPA buffer containing 0.1% SDS and supplemented with 1 × PIC and 50 µM NEM. After this, the cells were added with the VIVAbind™ SUMO matrix; the SUMO-unbound fractions or eluted SUMO-bound fractions were prepared according to the VIVAbind™ SUMO kit (viva bioscience). The isolated FA elution samples were used for western blotting analysis. The non-isolated whole cell lysate samples were prepared using the lysis buffer from the SUMO kit and followed the SUMO binding assay steps. The eluted SUMOylated proteins were reserved in 0.1% formic acid and submitted for mass spectrometry LC-MS/MS analysis. The data-dependent scanning acquisition was controlled by Xcalibur 2.1 software. The MS was run to obtain the spectra of digested peptides from the samples and the MS and MS/MS scans were searched against Uniprot database using SEQUEST algorithm (Thermo Fisher PD 1.4), where the database could provide theoretical spectra / computational calculated masses of peptides from the theoretical trypsin digested protein peptides (Birmingham Proteomics Unit, the Functional Genomics and Proteomics Laboratories, School of Biosciences, University of Birmingham).

### 4.10 Statistical analysis

All experiments were repeated independently at least three times and in each independent experiment, the data were pooled and averaged; the standard error (SEM) was calculated using Graphpad Prism (version 5). Figure legends list the *n* values and error bars (SEM) for each experiment. A one-way ANOVA post-hoc Tukey test was used for Fig. 1 (C, D and E: Ubc9 siRNA treatment), 4 (C and D: Ubc9 siRNA treatment) and 5 A for statistical difference between entire datasets. An unpaired two-tailed *t*-test was used for Fig. 1 (B, E: GA treatment), 2D, 3B and 4 (A and B: GA treatment) and Supplemental data S1 using Prism (version 5). p-value p < 0.0001 was reported as ***. Image J (FIJI 1.48 v) was used to count the time period of FA turnover. The time was noted for one FA to appear and disappear. This was performed for all the live-cell movies to calculate the mean turnover time of a FA. Image J was also used to calculate the mean number and size of a FA in these timelapse movies or images using automated identification of FAs following thresholding of the fluorescent images and particle tracking analysis. Each image threshold was adjusted first from the ‘image’ button. The upper and lower bar values for the threshold measure were noted and adapted for each image. Only focal adhesions (‘dots’) were selected with a red colour background (within the threshold tail). The image was in black and white. All the FAs ‘dots’ were made as areas of ‘white colour’. The image was made ‘binary’ in the ‘process’ button. This reversed the FAs colour to ‘black’ and the background to ‘white’. The image was selected from the ‘process’ with ‘binary’ to make it ‘watershed’, where the ‘black’ colour of FAs area was drawn boundaries manually according to the ‘original’ timelapse image. Counting was measured per cell. The image was ready to analyse ‘particles’ from the ‘analyse’ button. The size of the particle was set at 20 µm – Infinity (pixel units ticked) for the image. Each ‘particle’ was counted as ‘ellipse’ shape. The FAs were processed as ‘ellipse shaped’ only in the image. The mean number (count) and average size (µm^2^) were displayed as ‘Summary’ results. The speed of cell migration was measured using the plugins with the MTrackJ in Image J. For 1 cell movement, the tracking orbit of the cell was noted as a ‘new colour’ and each tracking was saved in ‘Summary result’ after completion.

### 4.11 Supplemental material

MDA-MB-231 cells were transfected with a GFP-FAK or a GFP-vinculin plasmid to detect focal adhesions on 2 mg/ml rat tail collagen I, these were done similarly to talin turnover assay. U2OS cells were grown on 0.2% gelatin coated glass coverslips. The cells were treated with 100 µM GA for 15 or 60 min and immunostained using mouse anti-vinculin monoclonal antibody, 1 mg/ml, MAB3574, Merck Millipore (1:100) or mouse anti-talin 1 monoclonal antibody.

## Conflict of interest

The authors declare that they have no conflicts of interest with the contents of this article.

## Author contributions

Z.Y. Huang designed and conducted the study, performed formal data analysis and wrote the original manuscript. D. Barker designed and conducted the experiments for Figs. 1A, C and 2A together with Z.Y. Huang. J. Gibbins contributed to resources, technical support and manuscript review. P. Dash contributed to project supervision, manuscript review and editing.

## Appendix A Supplementary material

**Figure S1. Inhibition of protein SUMOylation increases the number, size and turnover time of FAK or vinculin containing FAs in MDA-MB-231 cells; it also increases the number of talin or vinculin containing FAs in U2OS cells. Figure S1 A and B.** MDA-MB-231 cells were grown on top of 2 mg/ml collagen. 2 h of GA 100 µM treatment increased the mean number, size and turnover of FAK or vinculin containing FAs (data was presented as mean ± SEM; FAK: n = 6, individual replicated experiment, p < 0.0001***, vinculin: n = 4, individual replicated experiment, p < 0.0001***, p = 0.0014** for turnover time, two-tailed unpaired *t*-test). **Figure S1 C.** U2OS cells were grown on 0.2% gelatin-coated coverslips. Immunostaining of vinculin containing FAs were shown in the control or after 15 or 60 min of 100 µM GA treatment (scale bar=20 µm). 15 min of 100 µM GA treatment increased the mean number of vinculin containing FAs (n = 3, mean ± SEM, p = 0.0003***, two-tailed unpaired *t*-test); 1 h of 100 µM GA treatment increased the mean number of vinculin containing FAs (n = 3, mean ± SEM, p = 0.0017**, two-tailed unpaired *t*-test). **Figure S1 D.** U2OS cells were grown on 0.2% gelatin-coated coverslips. 15 min of 100 µM GA treatment increased the mean number of talin containing FAs (n = 3, mean ± SEM, p < 0.0001***, two-tailed unpaired *t*-test); 1 h of 100 µM GA treatment increased the mean number of talin containing FAs (n = 3, mean ± SEM, p = 0.0026**, two-tailed unpaired *t*-test). **Figure S2. Vinculin and filamin-A SUMOylation. A.** The isolated FAs with the SUMO binding assay showed that vinculin could be SUMOylated within the FAs (n = 3). **B.** Validation of filamin-1 SUMOylation. Filamin-1 at 280 kDa could be SUMOylated in MDA-MB-231cells (n = 3). **Table S1. The prediction of SUMO modification sites in talin-1.** Talin-1 SUMOylation predicted sites using SUMOplot: the lysine positions with score above 0.5 were highlighted in red. GPS SUMO programme showed both SUMOylation predictions and SUMO interactions SIMs of talin-1 SUMOylation. The FASTA sequence of talin-1 was inputted and the medium threshold was used. The modified positions, modified peptides, score for the modification, cut-off and the type of SUMO modifications were presented. **Table S2. SUMOplot and GPS SUMO predictions in p53, actin, FAK and Rac1.** The modified positions, modified peptides, score for the modification, cut-off and the type of SUMO modifications were presented. These were used to compare the detection of talin-1 SUMOylation sites.
