# Supplementary material for: Talin is a substrate for SUMOylation in migrating cancer cells
Source: Exp Cell Res. 2018 Sep 15;370(2):417–25. doi: 10.1016/j.yexcr.2018.07.005 (PMC6117455; doi:10.1016/j.yexcr.2018.07.005)
Supplement: Supplementary file 1 — Figure S1. Inhibition of protein SUMOylation increases the number, size and turnover time of FAK or vinculin containing FAs in MDA-MB-231 cells; it also increases the number of talin or vinculin containing FAs in U2OS cells. Figure S1 A and B. MDA-MB-231 cells were grown on top of 2 mg/ml collagen. 2 h of GA 100 µM treatment increased the mean number, size and turnover of FAK or vinculin containing FAs (data was presented as mean ± SEM; FAK: n = 6, individual replicated experiment, p < 0.0001***, vinculin: n = 4, individual replicated experiment, p < 0.0001***, p = 0.0014** for turnover time, two-tailed unpaired t-test). Figure S1 C. U2OS cells were grown on 0.2% gelatin-coated coverslips. Immunostaining of vinculin containing FAs were shown in the control or after 15 or 60 min of 100 µM GA treatment (scale bar=20 µm). 15 min of 100 µM GA treatment increased the mean number of vinculin containing FAs (n = 3, mean ± SEM, p = 0.0003***, two-tailed unpaired t-test); 1 h of 100 µM GA treatment increased the mean number of vinculin containing FAs (n = 3, mean ± SEM, p = 0.0017**, two-tailed unpaired t-test). Figure S1 D. U2OS cells were grown on 0.2% gelatin-coated coverslips. 15 min of 100 µM GA treatment increased the mean number of talin containing FAs (n = 3, mean ± SEM, p < 0.0001***, two-tailed unpaired t-test); 1 h of 100 µM GA treatment increased the mean number of talin containing FAs (n = 3, mean ± SEM, p = 0.0026**, two-tailed unpaired t-test). Figure S2. Vinculin and filamin-A SUMOylation. A. The isolated FAs with the SUMO binding assay showed that vinculin could be SUMOylated within the FAs (n = 3). B. Validation of filamin-1 SUMOylation. Filamin-1 at 280 kDa could be SUMOylated in MDA-MB-231cells (n = 3). Table S1. The prediction of SUMO modification sites in talin-1. Talin-1 SUMOylation predicted sites using SUMOplot: the lysine positions with score above 0.5 were highlighted in red. GPS SUMO programme showed both SUMOylation predictions and SUMO interacti [file mmc1.docx]

**SUPPLEMENTARY DATA**

**FIGURE S1**


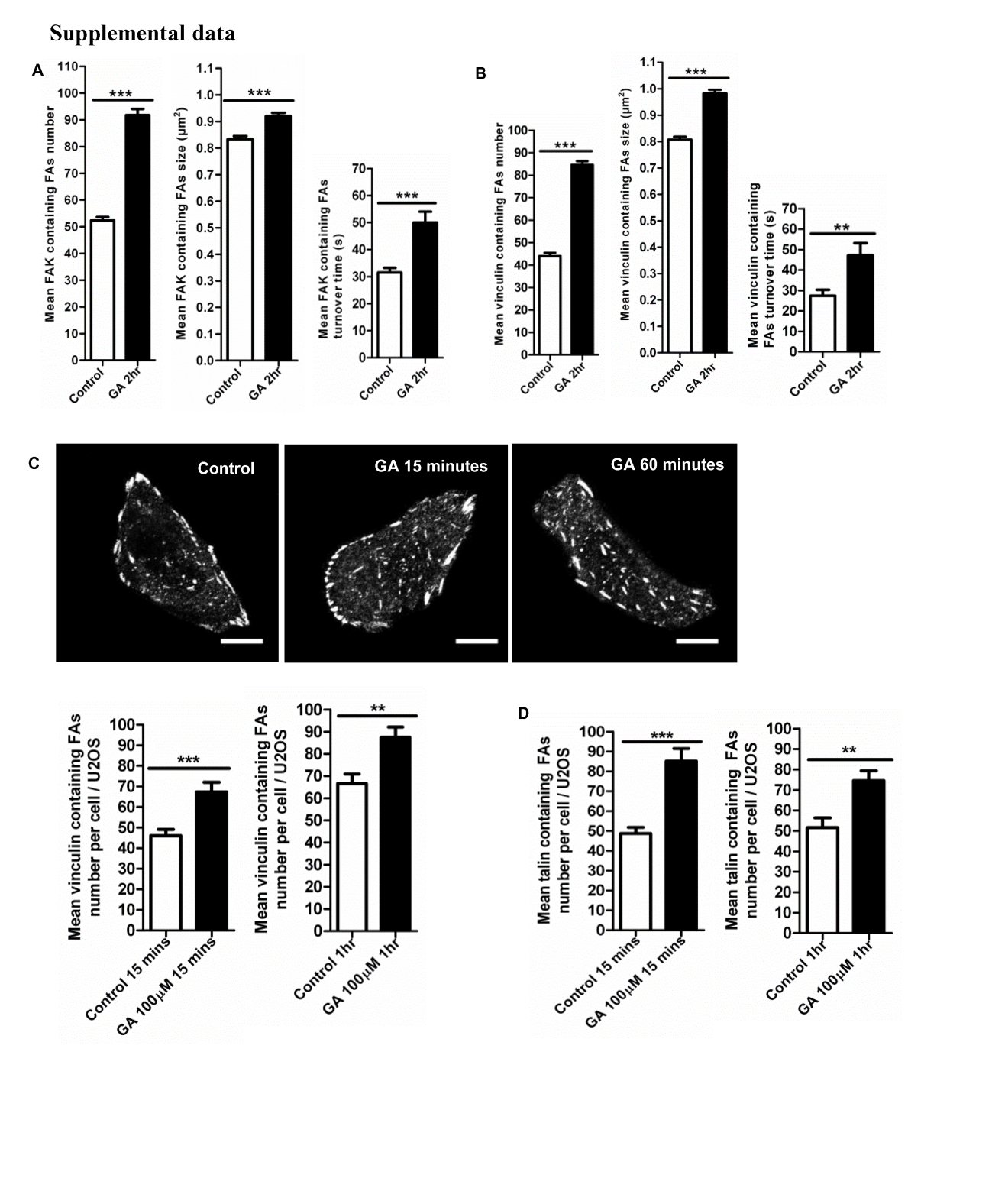


**FIGURE S2**


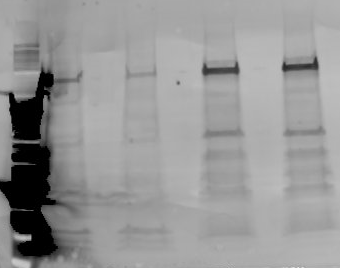


**Elution fraction**

**Unbound fraction**

Ctrl GA Ctrl GA (1hr)

SUMOylated Vinculin

130kDa

**Isolated FAs with SUMO binding assay**

**A**

**B**


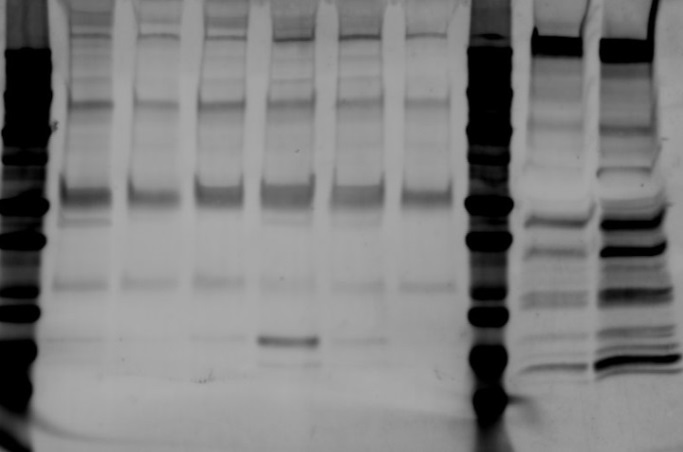


Ctrl 15 minutes

280

150

50

(kDa)

**HA IP: SUMO-2, IB: Filamin-1**

SUMOylated Filamin-1

**TABLE S1**

**SUMOplot^TM^ analysis programme (Abgent) predictions on the SUMOylation sites**

**Talin (protein ID:** [**Q9Y490**](http://www.uniprot.org/uniprot/Q9Y490)**): Motif B-K-x-D/E** (Motifs with probability score > 0.5)

| No. | Lysine Position | Peptide Sequence | Probability  Score |
| --- | --- | --- | --- |
| 1 | **K841** | DLVNA I**K**AD AEGES | 0.94 |
| 2 | **K2445** | LVACK V**K**AD QDSEA | 0.93 |
| 3 | K2063 | RLADV V**K**LG AASLG | 0.76 |
| 4 | K441 | QYNRV G**K**VE HGSVA | 0.67 |
| 5 | K1415 | GISQN A**K**NG NLPEF | 0.62 |
| 6 | K263 | AGFLD L**K**DF LPKEY | 0.56 |

**GPS SUMO programme predictions on talin protein peptide sequence**

| Position | Peptides | Score | Cut-off | Type |
| --- | --- | --- | --- | --- |
| **2445** | LLVACKVKADQDSEA | 44.828 | 36.625 | Sumoylation |
| **841** | SDLVNAIKADAEGES | 38.22 | 36.625 | Sumoylation |
| 300 | EAKVRYVKLARSLKT | 36.667 | 36.625 | Sumoylation |
| 1599 -1603 | GRAAMEP IVISA KTMLESA | 62.07 | 59.29 | SUMO Interaction |
| 2078 - 2082 | AEDPETQ VVLIN AVKDVAK | 61.629 | 59.29 | SUMO Interaction |
| 965 -969 | AVAEQIP LLVQG VRGSQAQ | 60.904 | 59.29 | SUMO Interaction |
| 396 - 400 | AQLIAGY IDIIL KKKKSKD | 60.757 | 59.29 | SUMO Interaction |
| 388 - 392 | QTTEGEQ IAQLI AGYIDII | 59.806 | 59.29 | SUMO Interaction |

**TABLE S2**

**SUMOplot p53 (Protein ID: P04637): Motif B-K-x-D/E** (Motifs with probability score > 0.8)

| No. | Lysine Position | Peptide Sequence | Probability Score |
| --- | --- | --- | --- |
| 1 | **K386** | HKKLM **FKTE** GPDSD | 0.85 |

**GPS SUMO Programme p53**

| Position | Peptides | Score | Cut-off | Type |
| --- | --- | --- | --- | --- |
| **386** | RHKKLMFKTEGPDSD | 17.901 | 16 | Sumoylation |
| 143 - 147 | QLAKTCP VQLWV DSTPPPG | 63.665 | 59.29 | SUMO Interaction |

**SUMOplot Actin, cytoplasmic 1 (Protein ID: P60709): Motif B-K-x-D/E** (Motifs with probability score > 0.8)

| No. | Lysine Position | Peptide Sequence | Score |
| --- | --- | --- | --- |
| 1 | **K284** | TFNSI **MKCD** VDIRK | 0.8 |
| 2 | **K68** | RGILT **LKYP** IEHGI | 0.8 |

**GPS SUMO programme actin**

| Position | Peptides | Score | Cut-off | Type |
| --- | --- | --- | --- | --- |
| **284** | TTFNSIMKCDVDIRK | 42.92 | 36.625 | Sumoylation |
| 103 - 107 | VAPEEHP VLLTE APLNPKA | 63.606 | 59.29 | SUMO Interaction |

**SUMOplot FAK (Protein ID: Q05397): Motif B-K-x-D/E** (Motifs with probability score > 0.8)

| No. | Lysine Position | Peptide Sequence | Probability  Score |
| --- | --- | --- | --- |
| 1 | **K152** | FFYQQ V**K**SD YMLEI | 0.93 |
| 2 | **K834** | KEERF L**K**PD VRLSR | 0.91 |

**GPS SUMO programme FAK**

| Position | Peptides | Score | Cut-off | Type |
| --- | --- | --- | --- | --- |
| **152** | NFFYQQVKSDYMLEI | 40.097 | 36.625 | Sumoylation |
| 903 | DSYNEGVKLQPQEIS | 39.886 | 36.625 | Sumoylation |
| 561 | VSSNDCVKLGDFGLS | 37.265 | 36.625 | Sumoylation |
| **834** | EKEERFLKPDVRLSR | 36.977 | 36.625 | Sumoylation |
| 121 | AHPPEEWKYELRIRY | 19.022 | 16 | Sumoylation |
| 267 - 271 | CALGSSW IISVE LAIGPEE | 73.562 | 59.29 | SUMO Interaction |
| 972 - 976 | LATVDET IPLLP ASTHREI | 67.784 | 59.29 | SUMO Interaction |
| 495 - 499 | GVITENP VWIIM ELCTLGE | 65.627 | 59.29 | SUMO Interaction |
| 487 - 491 | HPHIVKL IGVIT ENPVWII | 62.573 | 59.29 | SUMO Interaction |

**SUMOplot Rac1 (Protein ID: P63000): Motif B-K-x-D/E**

| Position | Peptides | Score | Cut-off | Type |
| --- | --- | --- | --- | --- |
| 7-11 | *MQAIKC VVVGD GAVGKTC | 59.593 | 59.29 | SUMO Interaction |
